# Bacterial Community Structure and Environmental Adaptation in the Endorhizosphere and Rhizosphere Soils of *Aeluropus sinensis* from Saline Lands Across Coastal and Inland Regions of China

**DOI:** 10.3390/microorganisms14010165

**Published:** 2026-01-12

**Authors:** Luoyan Zhang, Saiyu Han, Xiuxiu Guo, Lijie Wang, Yilin Fan, Xuejie Zhang, Shoujin Fan

**Affiliations:** Shandong Provincial Key Laboratory of Plant Stress Biology and Genetic Improvement, College of Life Science, Shandong Normal University, No. 1 Daxue Road, Jinan 250358, China; zhangluoyan@sdnu.edu.cn (L.Z.); 2022020852@stu.sdnu.edu.cn (S.H.); 622088@sdnu.edu.cn (X.G.); 2023027124@stu.sdnu.edu.cn (L.W.); 2024216017@stu.sdnu.edu.cn (Y.F.)

**Keywords:** *Aeluropus sinensis*, environmental adaptation, rhizosphere, bacterial community structure, yellow river delta

## Abstract

Bacterial communities in the rhizosphere and endorhizosphere of plants show distinct composition, function, and ecological roles during adaptation to diverse habitats. This study examines how rhizosphere and endophytic microbes associated with *Aeluropus sinensis*—a salt-excreting halophyte—contribute to its salt tolerance across saline-alkali environments. Microbial diversity and composition were analyzed via 16S rRNA gene amplicon sequencing. Soil physicochemical properties were measured to evaluate environmental effects. Linear regression assessed microbial–environment relationships, and co-occurrence networks identified key taxa and their adaptive strategies along environmental gradients. Soil salinity significantly affected rhizosphere bacterial diversity, with moderate levels increasing richness. Proteobacteria dominated both root and rhizosphere microbiomes across habitats. The endorhizosphere community strongly correlated with soil nutrients such as available phosphorus (AP) and total nitrogen (TN). Co-occurrence analysis reveals that chemoheterotrophic microbes in the *A. sinensis* rhizosphere employ distinct adaptive strategies across gradients, and ammonia-oxidizing bacteria (AOB) may support nitrogen cycling in the Yellow River Delta saline–alkaline ecosystem. This study underscores microbial adaptability in salt-tolerant grasses, demonstrating that comparing rhizosphere and endorhizosphere microbiomes in Poaceae under stress improves understanding of microbial functions in harsh environments.

## 1. Introduction

Plants growing in high-saline–alkali soil environments can mitigate the adverse effects of salt stress through well-documented physiological and biochemical mechanisms, including salt excretion, salt exclusion, and osmotic adjustment [1]. Furthermore, these plants are capable of modulating rhizosphere microbial community structures to create a micro-ecological environment that promotes their growth and development, thereby enhancing overall salt tolerance [2,3]. The Yellow River Delta serves as a representative coastal wetland ecosystem in China, characterized by elevated levels of soil salinity and alkalinity, limited nutrient availability, and an inherently fragile ecological environment [4]. Within this distinctive habitat, plant-associated rhizosphere bacterial communities perform essential functions, such as influencing plant growth and developmental processes, soil nutrient cycling, environmental adaptation, and restoring degraded ecosystems [5]. A comprehensive understanding of how halophytes facilitate their own survival and promote ecosystem development through the regulation of soil microbial composition is critical for elucidating the underlying mechanisms of plant salt tolerance and for formulating scientifically robust and practically effective ecological conservation strategies [6].

Poaceae plants possess well-developed root systems and exhibit strong environmental adaptability [7], allowing them to form close symbiotic relationships with a wide range of functional bacteria. The bacterial communities in their rhizosphere and endorhizosphere demonstrate distinct compositional differences, functional specialization, and ecological roles during the adaptation to diverse habitats [8]. The rhizosphere microbial community is primarily influenced by soil physicochemical properties such as pH, organic matter content, and salinity [9], reflecting a clear environmental filtering effect. Rhizosphere bacterial communities typically display higher α diversity than endorhizosphere communities, with dominant phyla including Proteobacteria, Firmicutes, Actinobacteriota, Bacteroidota, and Acidobacteriota [10,11,12]. In contrast, the endorhizosphere microbial community is mainly shaped by host plant factors such as root exudates, physiological structure, and genotype, leading to a more stable and functionally specialized microbial assemblage [13,14]. Patel and Archana [15] isolated numerous endorhizosphere bacterial strains with plant growth-promoting potential from Poaceae species, including maize, rice, sorghum (*Sorghum bicolor*), and wheat. These strains exhibit multiple beneficial traits, such as indole-3-acetic acid (IAA) production, nitrogen fixation, and phosphorus solubilization. A comprehensive analysis of the differences between rhizosphere and endorhizosphere microbial communities in representative Poaceae plants under adverse environmental conditions is essential for understanding the ecological functions and adaptive mechanisms of microorganisms in stress-prone habitats.

*Aeluropus sinensis* (Debeaux) Tzvelev, a perennial herb endemic to China and belonging to the genus *Aeluropus* within the Poaceae family, is widely distributed in saline–alkali soils along northern coastal and inland regions [4]. It can grow normally in soils with salt content as high as 1% [16]. This species exhibits strong vegetative propagation capacity and can stabilize sandy soils through dense stolons and well-developed root systems, contributing significantly to the ecological restoration of degraded coastal and salt lake margin ecosystems [17]. Previous studies have demonstrated that under mild salt stress conditions, *A. sinensis* effectively reduces rhizosphere soil pH while increasing key nutrient contents such as total nitrogen and available phosphorus [4,5,6]. These changes improve soil physicochemical properties and promote the enrichment and maintenance of rhizosphere bacterial diversity. Under moderate salt stress, however, rhizosphere bacterial diversity declines. Nevertheless, the bacterial diversity in the rhizosphere (Shannon index) remains significantly higher than in non-rhizosphere soils. The dominant phyla include Proteobacteria, Actinobacteria, and Chloroflexi, with major ecological functions centered on chemoheterotrophy and aerobic metabolism [18]. Although existing research has preliminarily elucidated the potential roles of microbial communities in the salt stress response of *A. sinensis*, it remains unclear how rhizosphere and endorhizosphere bacteria interactively influence plant adaptation under multiple environmental variations dominated by salinity stress, such as differences in temperature, rainfall, and pH.

China possesses diverse saline–alkali land environments, characterized by high salinity levels, low soil fertility, and ecologically fragile conditions [19]. Geographically, these can be categorized into coastal wetland saline–alkali land and inland saline–alkali land. In recent years, advances in high-throughput sequencing technology have enabled extensive investigations into the community structure, functional diversity, and ecological impacts of rhizosphere bacteria in the Yellow River Delta. These studies have revealed complex regulatory mechanisms driven by multiple factors, including host plant species, soil properties, and biological invasions. Furthermore, environmental variables such as salinity, hydrology, rainfall, heavy metal contamination, and petroleum pollution have been shown to influence the diversity and composition of soil bacterial communities in this region [20,21,22,23]. This study focuses on how rhizosphere and endophytic microorganisms associated with *A. sinensis*—a salt-excreting halophyte—contribute to its salt tolerance across different types of saline–alkali land environments.

## 2. Materials and Methods

### 2.1. Sampling Sites

**Figure 1:**
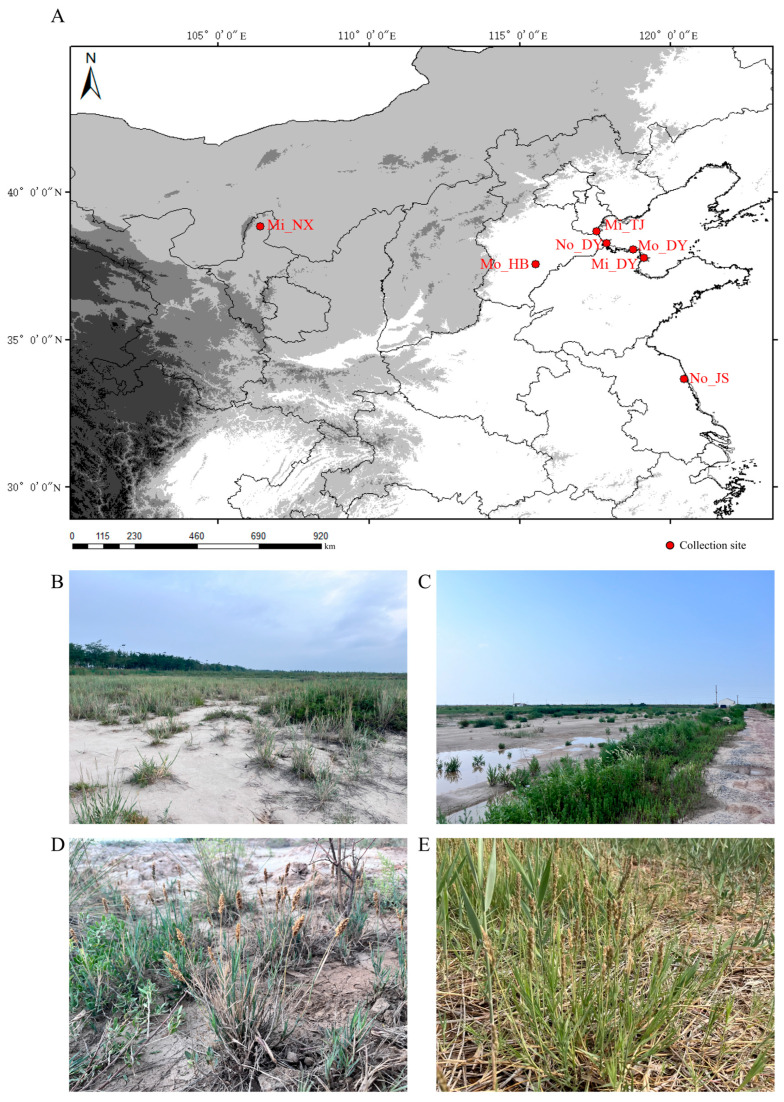
Schematic diagram showing the sampling sites of *Aeluropus sinensis* from saline lands across coastal and inland regions of China. (**A**) This study included typical saline–alkali areas from eastern coastal and selected inland regions of China, representing two major ecological types. Based on their geographical location and salinity classification, these sites were designated as the coastal type covers wetlands in Dongying (Mo_DY, Mi_DY, No_DY), Tianjin Binhai (Mi_TJ), and Yancheng (No_JS); the inland type includes regions in Shizuishan (Mi_NX) and Hengshui (Mo_HB). Habitat characteristics and Associated Plant Species at the Mi_NX (**B**,**D**) and Mo_DY (**C**,**E**) Sampling Site.

This study encompassed typical saline–alkali lands across eastern coastal and selected inland regions of China, representing two major ecological types [20]. The first type consists of coastal wetland zones located in Dongying City, Shandong Province (DY), Binhai New Area, Tianjin (TJ), and Yancheng City, Jiangsu Province (JS) (Figure 1 and Table 1). These areas are situated within the Yellow River Delta and along the coasts of the Bohai Sea and Yellow Sea, characterized by typical tidal flat wetlands and coastal saline–alkali soils. The second type comprises inland saline–alkali zones represented by Shizuishan City in Ningxia Hui Autonomous Region (NX) and Hengshui City in Hebei Province (HB), which typify the inland saline soil ecosystems found in the arid and semi-arid regions of northern China. These sampling sites span multiple ecological habitats where *A. sinensis* is distributed, reflecting its broad adaptability to diverse saline–alkali environments. This study adopts the classification system for inland and coastal tidal flat soils in China as outlined in the book “Chinese Saline Soil” [24]. Soil salinity was determined for all collected samples using electrical conductivity (EC, unit: mS/cm) as an indicator and categorized into three levels: no salt (No): EC < 2.0; low salt (Mi): 2.0 ≤ EC < 15.0; medium salt (Mo): EC ≥ 15.0. A total of seven sampling sites across five geographic regions were selected in this study. Based on their geographical location and salinity classification, these sites were designated as Mo_DY, Mo_HB, Mi_NX, Mi_DY, Mi_TJ, No_JS, and No_DY (Figure 1 and Table 1).

### 2.2. Soil Sample Collection

All sampling was conducted between July and August 2024 using the five-point sampling method. Within each 4 m^2^ plot (2 m × 2 m), sampling points were established at the four corners and at the central intersection of the diagonals within each sample plot. At each point, roots of *A. sinensis* were collected, with three individual root samples combined to form a composite sample [20]. Rhizosphere soil and plant tissue samples were collected in the field and transferred to the laboratory using liquid nitrogen tank.

Rhizosphere soil samples (Rh): Roots were carefully excavated using a sterilized sampling shovel, and large soil clumps adhering to the roots were manually removed by shaking. The roots, along with the tightly adhering rhizosphere soil, were placed into sterile bags and labeled. In a laminar flow hood, non-rhizosphere soil was further removed by shaking, leaving only the soil layer adhering to the root surface within approximately 1 mm, which was defined as rhizosphere soil. The root samples were then transferred into sterile 50 mL centrifuge tubes containing 20 mL of sterile 10 mM PBS solution and shaken at 120 rpm at room temperature for 20 min. Using sterile forceps, the roots were removed from the tubes, and the remaining suspension was centrifuged at 6000× *g* for 20 min at 4 °C to collect the rhizosphere soil. In the laboratory, the Rh samples were divided into two parts: one part was stored at 4 °C for soil physicochemical property analysis, and the remaining part was frozen at −80 °C for total genomic DNA extraction [25].

Root samples (Ri): In a laminar flow hood, the collected roots were subjected to surface sterilization. Briefly, the samples were immersed in 75% ethanol for 1 min, followed by a 5–10 min treatment in a 2.5% NaClO solution, and subsequently rinsed 2–3 times with sterile distilled water. Excess surface moisture was removed using sterile filter paper. The sterilized roots were then placed into sterile 10 mL EP tubes, labeled, and either used immediately for experiments or flash-frozen in liquid nitrogen and stored at −80 °C for DNA extraction and preservation [26].

### 2.3. Physical and Chemical Property Measurements

Electrical conductivity (EC) was measured with a DDSJ-308A conductivity meter (Shanghai Yidian Scientific Instrument Co., Ltd., Shanghai, China) in a 1:5 (*w*/*v*) soil-to-water solution, based on Li et al. [27]. Soil pH was determined using a pH meter with a 1:2.5 (*w*/*v*) soil-to-water suspension, following the procedure described by Kuang et al. [20]. Soil moisture content (SM) was measured using the oven-drying method, as outlined by Liu et al. [28]. Total nitrogen (TN) was analyzed using the semi-micro Kjeldahl digestion method, according to Gonze et al. [29]. Available phosphorus (AP) was extracted with 0.5 M NaHCO_3_ and subsequently quantified through molybdenum-antimony colorimetry, following Zhou et al. [30]. All soil analyses were conducted in triplicate to ensure reliable and accurate measurements. Altitude refers to the height difference from mean sea level (MSL).

### 2.4. Extraction and Sequencing of Soil Total DNA

Sample submission for sequencing was performed on September 2024. In this study, a no-template control was incorporated during the DNA extraction process to monitor for potential contamination and ensure the integrity and reliability of downstream amplification results. Total DNA was extracted from soil samples using the E.Z.N.A.^®^ Soil DNA Kit (DNA extraction kit, Omega Bio-Tek, Norcross, GA, USA). For each sample, three replicates were prepared and verified via 1% agarose gel electrophoresis. The diluted DNA was used as a template for PCR amplification. Universal primers 338F (5′-ACTCCTACGGGAGGCAGCAG-3′) and 806R (5′-GGACTACHVGGGTWTCTAAT-3′) were employed to amplify the V3–V4 hypervariable regions of the bacterial 16S rRNA gene. The PCR reaction mixture was composed of: 4 μL of 5 × TransStart FastPfu buffer, 2 μL of 2.5 mM dNTPs, 0.8 μL of forward primer (5 μM), 0.8 μL of reverse primer (5 μM), and 0.4 μL of TransStart FastPfu DNA polymerase. A total of 10 ng of template DNA was added, and the final volume was adjusted to 20 μL with nuclease-free water. The thermal cycling program included an initial pre-denaturation at 95 °C for 10 min, followed by 30 cycles of denaturation at 95 °C for 30 s, annealing at 55 °C for 30 sec, and extension at 72 °C for 1.5 min. This was followed by a final extension step at 72 °C for 10 min, after which the reaction was maintained at 4 °C. Amplification was performed using an ABI GeneAmp^®^ 9700 thermal cycler (Thermo Fisher Scientific, MA, USA). PCR products were confirmed via 0.7% agarose gel electrophoresis. To ensure representativeness, five PCR replicates from each soil sample were pooled to form a composite sample, which was then sequenced on the Illumina NovaSeq 6000 platform at Majorbio Bio-Pharm Technology Co., Ltd. (Shanghai, China). The sequencing data has been uploaded to the NCBI database, with the accession number PRJNA1273014.

### 2.5. Data Analysis

We analyzed the physicochemical properties of *A. sinensis* rhizosphere soil using the “vegan (Version 2.7)” package in R and assessed inter-group differences in these properties via *t*-tests. The α-diversity indices, including the Chao1 index for microbial richness and the Shannon index for diversity, were calculated using the diversity and specnumber functions in the “vegan” package. Differences in α-diversity indices among groups were statistically evaluated using *t*-tests [31]. To assess significant differences in microbial community composition across different sampling sites of *A. sinensis*, principal coordinate analysis (PCoA) and permutational multivariate analysis of variance (PERMANOVA) were conducted based on Bray–Curtis distances using the “vegan” and “GUniFrac (Version 1.9)” packages. A linear regression model was employed to examine the correlations between rhizosphere and endorhizosphere bacterial communities and environmental factors. Redundancy analysis (RDA) was performed using the “vegan” package in R to investigate the relationships between key environmental variables and the relative abundance of microbial phyla.

Co-occurrence networks were constructed using CoNet v.1.0.6 beta [32], incorporating both Spearman’s correlation and Kullback–Leibler dissimilarity (KLD) measures [33]. Bacterial OTUs that appeared in less than 20% of the sampling sites and exhibited a total relative abundance below 0.001% across all rhizosphere samples [34] were excluded from the network analysis. The dissimilarity threshold to the maximum value of the KLD matrix and Spearman’s correlation threshold to 0.7. The adjacency matrix is transformed into an adjacency list of the igraph network using igraph (Version 2.0) in R, and the Louvain algorithm is employed with igraph and WGCNA (Version 1.73) to obtain modules. To investigate the associations between microbial modules and environmental factors, we calculated the module eigengene E, defined as the first principal component of each module [35], for the dominant modules in the rhizosphere networks. Subsequently, Spearman’s rank correlation test was employed to assess the correlations between these eigengenes and environmental variables (EC, pH, AP and TN). Network visualizations were constructed using Cytoscape 3.10.0. Functional prediction of the filtered modules was performed using Faprotax (Version 1.2.10) on the Majorbio Data Analysis Platform (https://v.majorbio.com/, accessed on 10 January 2025) to elucidate potential mechanisms underlying environmental tolerance in *A. sinensis* rhizosphere bacteria.

### 2.6. Isolation, Purification and Identification of Salt-Tolerant Bacteria in Rh

In this study, salt-tolerant bacteria were screened from rhizosphere soil samples collected at the Mo_DY site using the enrichment culture method. Rhizosphere soil was suspended in 50 mL of sterilized high-salt LB liquid medium and incubated on a shaker at a constant temperature of 30 °C and 150–250 rpm for 72 h. Following enrichment, the bacterial suspension was serially diluted to 10^−^^3^, 10^−4^, and 10^−5^ in a sterile laminar flow cabinet. Then, 100 µL of each dilution was evenly spread onto the surface of high-salt LB solid medium and incubated at 37 °C for 48 h. During this period, colony growth was monitored regularly, and morphological characteristics—including shape, size, and color—were documented. Distinct single colonies were selected based on visible differences in morphology, purified through repeated streaking on fresh media, and ultimately used for further analysis.

Colonies exhibiting distinct morphological differences were selected and subjected to 16S rRNA gene amplification using the universal primers 27F (5′-AGAGTTTGATCMTGGCTCAG-3′) and 1492R (5′-TACGGYTACCTTGTTAYGACTT-3′). The PCR amplification was performed according to the reaction system and thermal cycling conditions described by Haas et al. [36]. Following amplification, the PCR products were electrophoretically analyzed on a 1% agarose gel to confirm successful amplification. Successfully amplified products were submitted to BGI for purification and Sanger sequencing. The resulting 16S rRNA gene sequences were then used for similarity searches against the appropriate databases on NCBI (https://www.ncbi.nlm.nih.gov/, accessed on 10 January 2025) to identify closely related bacterial species.

### 2.7. Maize Cultivation and Physicochemical Indicators Determination

Maize seeds (Zhengdan 958) were germinated under controlled conditions. Uniformly growing seedlings were then selected for pot experiments, with three plants per pot. The bacterial irrigation experiment was initiated when the maize plants reached the three-leaf stage. Four treatment groups were established: Control A (no bacteria, no salt stress), Control B (salt stress, no bacteria), Experimental Group A (salt stress + bacterial inoculation), and Experimental Group B (no salt stress + bacterial inoculation). In the experimental groups, each pot was inoculated with a distinct bacterial strain, and 200 mL of bacterial suspension was applied to each pot. In the control experiment, Hogland nutrient solution was applied to each pot; in the salt stress treatment, a modified Hogland nutrient solution supplemented with 200 mM NaCl was used to impose salinity stress. All treatments were replicated to ensure consistent bacterial inoculation across samples. The maize seedlings were grown in a controlled environment with a 16 h light (600 μmol m^−2^ s^−1^)/8 h dark photoperiod at 25 °C and watered twice weekly with half-strength Hoagland’s nutrient solution. Plant growth parameters were assessed after 21 days of treatment.

Three biological replicates per group were selected for the measurement of growth and physiological parameters. Chlorophyll content was quantified using the ethanol-acetone extraction method [37], while proline content was determined via the acid ninhydrin colorimetric assay [38]. Data were recorded and processed using Microsoft Excel, and further statistical analysis and graphical visualization were conducted with the ggplot2 (Version 4.0) and ggpubr (Version 4.0) packages in R. One-way analysis of variance (ANOVA) was performed to assess inter-group differences, followed by Duncan’s multiple range test for post hoc pairwise comparisons. Statistical significance was defined as *p* < 0.05.

## 3. Results

### 3.1. Environmental Variables of Sampling Sites

**Figure 2:**
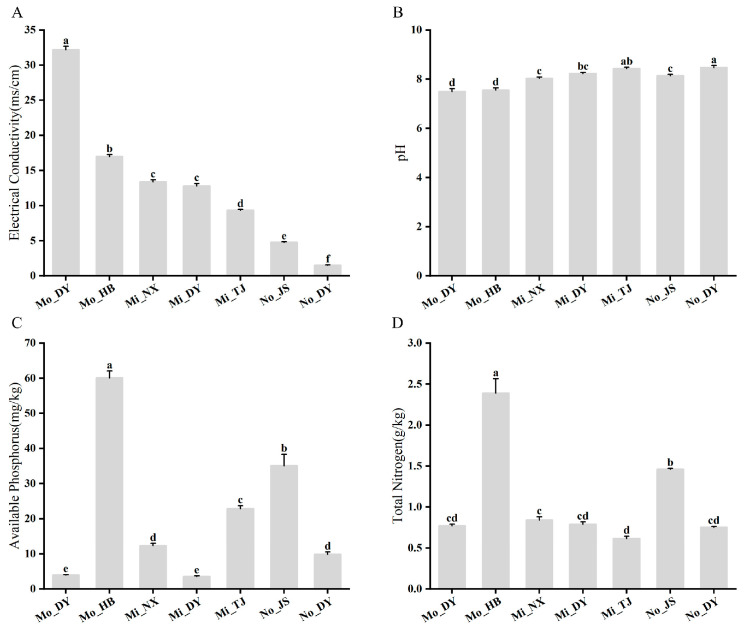
Differences in physicochemical properties of rhizosphere soils of *A. sinensis* at seven sampling sites. (**A**) The electrical conductivity (EC) value of the soil salinity in the sample. (**B**) The pH value of the soil sample. (**C**) The available phosphorus content in the soil sample. (**D**) The total nitrogen content in the soil sample. Lowercase letters (a, b, c, d, e, f) denote statistically significant differences (*p* < 0.05) in α-diversity indices among samples.

The results indicated that all sampled soils were slightly alkaline, with pH values ranging from 7.4 to 8.5 (Figure 2A,B); however, significant variations were observed in terms of electrical conductivity (EC), available phosphorus (AP), and total nitrogen (TN) content (Figure 2A,C,D). The highest pH levels were recorded at No_DY (8.43 ± 0.05) and Mi_TJ (8.46 ± 0.08) (Figure 2B). The maximum soil EC value (32.16 ± 0.49 mS/cm) was observed at Mo_DY, whereas the lowest EC values were found at No_JS (4.76 ± 0.08 mS/cm) and No_DY (1.5 ± 0.0 mS/cm) (Figure 2A). Additionally, Mo_HB exhibited the highest concentrations of both AP (60.06 ± 1.99 mg/kg) and TN (2.38 ± 0.17 g/kg) (Figure 2C,D).

### 3.2. Bacterial Community Composition and Structure of Endorhizal Tissue and Rhizosphere Soil

The Mo_DY_Rh and Mi_DY_Rh samples exhibited the highest OTU counts, Chao and Shannon index (*p* < 0.05), whereas the No_JS_Rh sample showed relatively lower α-diversity values (Table 2). Notably, this site displayed the highest Simpson index (*p* < 0.05), while the Simpson indices of other sites were comparatively similar. Analysis of bacterial community composition within endorhizal tissues indicated that Mo_HB_Ri had the greatest OTU richness and Chao1 index (*p* < 0.05), with the remaining six sites showing comparable levels. High Shannon index values were observed for Mo_HB_Ri, Mi_DY_Ri, and Mi_NX_Ri (*p* < 0.05), indicating greater bacterial diversity in the roots at these locations. The No_DY_Ri site exhibited the highest Simpson index (*p* < 0.05), implying low community evenness, a trend consistent with that observed in the rhizosphere bacterial communities.

**Figure 3:**
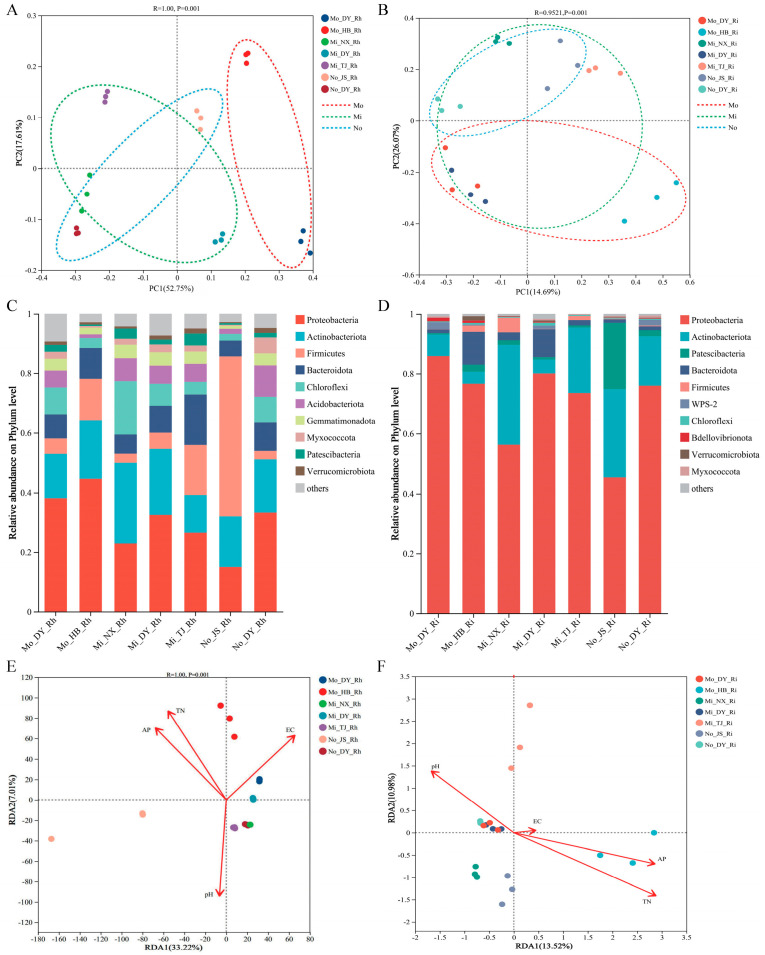
Bacterial community composition and structure of rhizosphere soil and endorhizal tissues. (**A**) PCoA analysis of bacterial communities in rhizosphere soils of *A. sinensis*. (**B**) PCoA analysis of bacterial communities in endorhizal tissues. (**C**) Phylum-level community composition of bacteria in rhizosphere soil. (**D**) Phylum-level community composition of bacteria in endorhizal tissues. Ordination Plot of Redundancy Analysis (RDA) results to determine the relationship between bacterial community structure and soil environmental factors. (**E**) The RDA results of rhizosphere soil. (**F**) The RDA results of endorhizal tissues. EC: electrical conductivity; TN: total nitrogen; AP: available phosphorus.

In the rhizosphere bacterial analysis (Figure 3A), the first two principal coordinates (PC1 and PC2) accounted for 70.36% of the total variation, indicating a strong representation of the overall data structure. The distribution pattern revealed a clear separation among samples from different salinity environments. Specifically, samples from the medium-salinity sites (Mo_DY_Rh and Mo_HB_Rh) clustered predominantly along the positive direction of PC1, whereas those from low- and non-salinity regions were mainly located along the negative axis and central region, with some degree of overlap. In contrast, the PCoA analysis of endophytic bacterial communities (Figure 3B) explained 59.56% of the total variation through PC1 and PC2. Unlike the rhizospheric communities, no clear salinity-related clustering pattern was observed within the root interior. Notably, the three Dongying samples (Mo_DY_Ri, Mi_DY_Ri, and No_DY_Ri) clustered closely together, indicating a high degree of similarity in their endophytic bacterial composition. Conversely, Mo_HB_Ri, characterized by high soil salinity and nutrient levels, was distinctly separated from the other sampling sites.

The 10 most abundant bacterial phyla were selected for further examination of the rhizosphere and endorhizosphere bacterial community composition (Figure 3C,D). The dominant bacterial groups in the rhizosphere soil primarily included Proteobacteria, Actinobacteriota, Firmicutes, and Bacteroidota. In the medium-salinity sites (Mo_DY_Rh and Mo_HB_Rh), the relative abundance of Proteobacteria reached 38% and 45%, respectively, which were significantly higher than those in low-salinity and non-saline environments. In the low-salinity sites (Mi_DY_Rh, Mi_NX_Rh, and Mi_TJ_Rh), Actinobacteriota exhibited relative abundances of 27%, 13%, and 22%, all of which were higher than those observed in the medium-salinity sites. In the non-saline samples (No_DY_Rh and No_JS_Rh), the abundance of Firmicutes increased markedly, particularly in No_JS_Rh, where it reached 54%, substantially exceeding the levels found in both medium- and low-salinity samples. Notably, the relative abundance of Proteobacteria reached 86% and 77% in the middle-salinity sites (Mo_DY_Ri and Mo_HB_Ri), which was markedly higher than in endophytic samples from other salinity levels, as well as in rhizospheric samples from the same middle-salinity sites. Furthermore, Patescibacteria accounted for 22% of the bacterial community in the no-salinity site (No_JS_Ri), a significantly higher proportion than in other sites.

### 3.3. Associations Between Bacterial Communities and Environmental Factors

At the OTU level, RDA revealed that the first and second axes explained 33.22% and 7.01% of the variation in bacterial community composition within the rhizosphere soil of *A. sinensis*, respectively, with a cumulative variance explanation of 40.23% (Figure 3E). The structure of rhizospheric bacterial communities was significantly influenced by several soil physicochemical factors, including EC, pH, AP, and TN, indicating that these environmental variables are closely associated with the composition and spatial distribution of rhizosphere microbial communities. In contrast, the RDA patterns for endophytic bacterial communities differed markedly: at the OTU level, the two axes together explained only 10.98% of the variation (Figure 3F). Endophytic community structure was primarily shaped by soil AP and TN content, followed by soil pH, with minimal influence from soil salinity.

**Figure 4:**
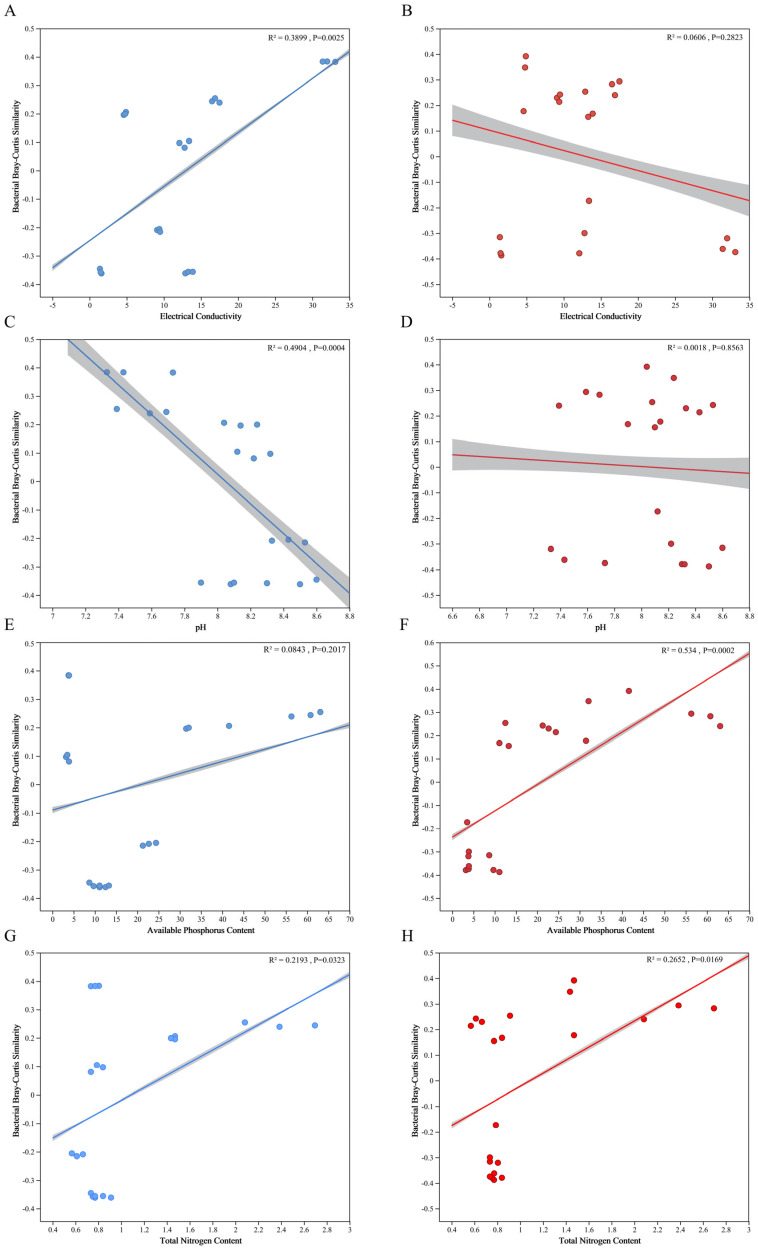
Distance-decay relationships of bacterial communities associated with environmental variations, based on Bray–Curtis similarity. Bacterial community similarity of rhizosphere soils plotted against soil electrical conductivity (**A**), pH (**C**), available phosphorus (**E**) and total nitrogen (**G**). Bacterial community similarity of endorhizal tissues plotted against soil electrical conductivity (**B**), pH (**D**), available phosphorus (**F**) and total nitrogen (**H**).

The rhizosphere bacterial community exhibited a significant positive correlation with soil electrical conductivity (EC), an indicator of soil salinity (R^2^ = 0.39, *p* < 0.01) (Figure 4A), and a significant negative correlation with soil pH (R^2^ = 0.49, *p* < 0.01) (Figure 4C). However, no significant distance-decay similarity was detected in relation to soil nutrient elements (Figure 4E). In contrast, the regression results for intraradical bacterial communities differed markedly from those of the rhizosphere: the intraradical communities showed significant positive correlations with soil AP and TN (R^2^ = 0.53, *p* < 0.01; R^2^ = 0.27, *p* < 0.01) (Figure 4F,H), but no significant relationships were observed with soil EC or pH.

### 3.4. Co-Occurrence Network Analysis of Bacterial Communities in the Medium-Salinity Soil

**Figure 5:**
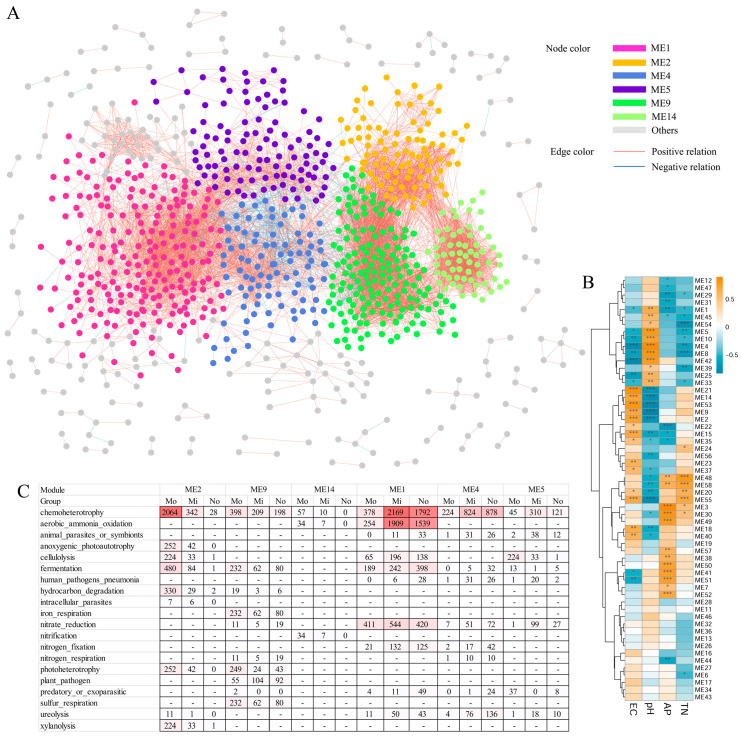
(**A**) Co-occurrence networks of rhizosphere microbial communities in A. sinensis. Networks are colored by modules, the six largest modules are marked in different colors, while the rest are labeled in gray. (**B**) Spearman’s correlations between modules and environmental variables (EC, pH, AP and TN) in global networks of rhizosphere. The *p*-values are presented as asterisks above the box plots. *** indicates *p*-value < 0.005, ** indicates *p*-value < 0.01, * indicates *p*-value < 0.05. (**C**) The Faprotax functional enrichments of the screened modules. The color scale reflects the extent of sample enrichment for potential functional traits.

Co-occurrence networks of bacterial communities in rhizosphere soils were constructed at the OTU level (Figure 5A and Table S1). A total of 8623 co-occurrence relationships (edges) among 968 nodes were identified in this study, and the six most substantial modules are displayed and marked in Figure 5. This study revealed, through statistical analysis, that modules 2, 9, and 14 exhibited a positive correlation with EC, while modules 1, 4, and 5 showed a positive association with Ph and soil nutrient element content. Module ME9 consists of 166 nodes, Faprotax-based functional prediction analysis indicated that the OTUs within this module are associated with metabolic processes including chemoheterotrophy, fermentation, iron respiration, photoheterotrophy, and sulfur respiration in middle-salt microbial communities (Figure 5B,C). Module ME2 comprises 93 OTUs and is predominantly associated with functional processes including chemoheterotrophy, anoxygenic photoautotrophy, cellulolysis, hydrocarbon degradation, and xylanolysis in the middle-salt community (Figure 5B,C). The pH-associated module ME4 in the salt-free group is primarily involved in metabolic processes including chemoheterotrophy, nitrate reduction, nitrogen fixation, and ureolysis (Figure 5B,C).

### 3.5. Isolation of Salt-Tolerant Bacterial Strains and Irrigation Experiments on Maize

**Figure 6:**
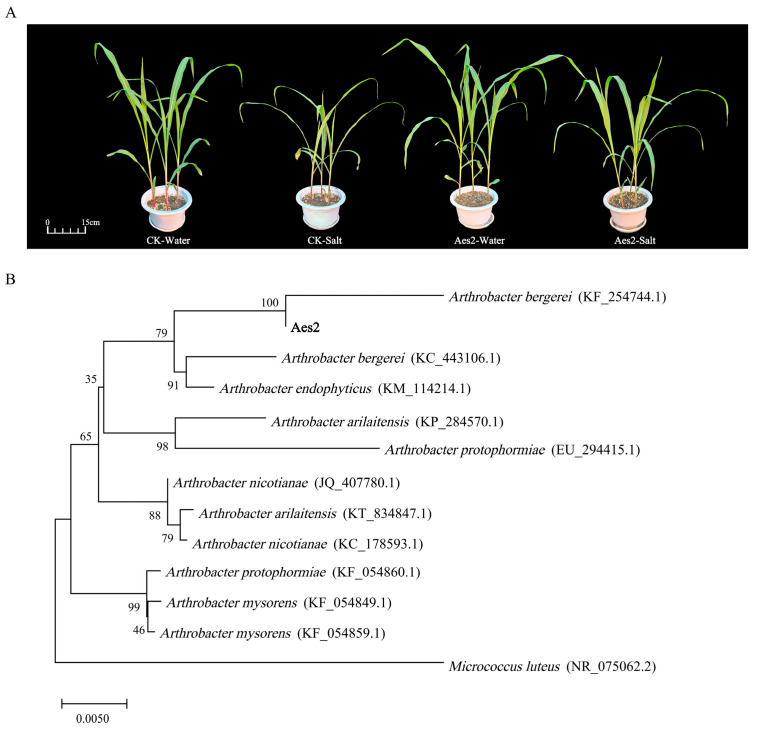
The morphological performance of maize seedlings inoculated with Aes2 was examined after 21 days of salt stress (**A**), CK-water: No salt treatment and no bacterial inoculation; CK-salt: Salt treatment without bacterial inoculation; Aes2-water: Bacterial inoculation without salt treatment; Aes2-salt: Salt treatment combined with bacterial inoculation. The bolded font highlights the information of the selected salt-tolerant strains. (**B**) The phylogenetic position of strain Aes2. The phylogenetic analysis indicated that Aes2 had the highest sequence similarity with *Arthrobacter bergerei* (KF_254744.1).

A total of 11 bacterial isolates were obtained from the rhizosphere soil of *A. sinensis* collected from the Mo_DY site. All bacterial strains were cultured on LB medium, and their colony morphologies were observed (Figure S2). The isolates were numbered, and their colony characteristics, including morphology, color, and size. To identify salt-tolerant strains, the effects of each isolate on the growth and physiological-biochemical parameters of corn were evaluated under salt stress conditions (Figure S3). No significant differences in plant fresh weight were observed after inoculation with Aes1, Aes4, or Aes5 under salt stress. Similarly, no significant differences in dry weight were detected in plants treated with Aes2, Aes4, Aes5, Aes6, Aes8, or Aes11. Chlorophyll content remained unchanged in plants inoculated with Aes1, Aes7, or Aes10, while proline content showed no significant variation in those treated with Aes7 or Aes11. The overall impact of each strain on corn salt tolerance was assessed using a membership function analysis, which revealed that strain Aes2 exerted a comprehensive positive effect on salt tolerance (Table 3). Furthermore, the morphological performance of corn seedlings inoculated with Aes2 was examined after 21 days of salt stress (Figure 6A and Figure S2).

The sequences of the strains obtained by 16S rRNA gene sequencing were uploaded to the NCBI database, and the BLAST tool (Version 1.4.0) was used to compare them with the known sequences with high similarity in the database. To further clarify the phylogenetic position of strain Aes2, 16S rRNA gene reference sequences with high similarity to it were selected. Based on the comparison results, multiple sequence alignment was performed on the obtained sequences using MEGA 7.0 software, and a phylogenetic tree was constructed. *Micrococcus luteus* (NR_075062.2) was selected as the outgroup. The results of the phylogenetic analysis indicated that Aes2 had the highest sequence similarity with *Arthrobacter bergerei* (KF_254744.1), and the two were clustered in the same evolutionary branch (Figure 6B).

## 4. Discussion

### 4.1. Diversity of Soil Physicochemical Properties

*A. sinensis* is a perennial herb endemic to northern China, commonly found in coastal and inland saline soils. This study collected soil samples from multiple regions to examine how environmental factors, especially soil physicochemical properties, shape microbial communities in the rhizosphere and root endosphere of *A. sinensis*. Results revealed significant variation in salinity, pH, and nutrient composition across sites. The Yellow River Delta, a highly saline coastal wetland, was represented by three sites with distinct salinity levels. No direct correlation was observed between soil electrical conductivity (EC) and pH: Mo_DY had the highest EC (32.16 ± 0.49 mS/cm) but low pH (7.49 ± 0.12), while No_DY had the lowest EC (1.5 ± 0.0 mS/cm) but higher pH (8.43 ± 0.05) (Figure 1A,B). This decoupling may result from factors such as hydrology, precipitation, heavy metal contamination, and petroleum pollution. Previous studies show that *A. sinensis* can secrete allelochemicals under high salinity, potentially reducing soil salinity and alkalinity. At Mo_HB, a distinct nutrient enrichment pattern emerged (Figure 1C,D): despite high salinity, TN and AP levels were significantly higher than at other sites. Nutrient enrichment can have dual effects on root-associated bacterial communities. Optimal TN and AP levels enhance bacterial metabolic activity and functional diversity, promoting nitrogen-cycling taxa [39]. However, long-term excess may cause nutrient saturation, favoring dominant taxa and suppressing others, leading to simplified community structure and reduced α-diversity [40].

### 4.2. Bacterial Composition and Diversity Differences in Rhizosphere Soil

Variations in bacterial richness and diversity across rhizosphere soils indicate that environmental factors, especially soil physicochemical properties, strongly influence the rhizosphere microbial community of *A. sinensis*. This study shows that salinity plays a key role in shaping bacterial diversity. Observed OTUs, Chao1, and Shannon indices were significantly higher under medium and low salinity than in non-saline conditions (Table 2), suggesting moderate salinity enhances rhizosphere bacterial diversity. Linear regression revealed a significant positive correlation between the rhizosphere bacterial community and soil electrical conductivity (R^2^ = 0.39, *p* < 0.01) (Figure 2A). While some studies report negative effects of salinity on bacterial diversity [41,42] and Zhao et al. [43] found lower richness and α-diversity in saline soils in the YRD, others show that in extreme saline lakes and coastal wetlands, increased salinity can enrich halotolerant bacteria and enhance β-diversity [44]. These discrepancies suggest salinity’s impact depends on microhabitat characteristics, functional redundancy, and niche adaptation [45]. We hypothesize that the salinity range in this study (up to 15.0 mS/cm) represents moderate salt stress for *A. sinensis*, under which microbial diversity may increase. Soil pH is a major driver of rhizosphere bacterial diversity and structure. High pH often inhibits microbial growth and reduces diversity. Our results confirm this, showing a significant negative correlation between rhizosphere diversity and soil pH (R^2^ = 0.49, *p* < 0.01) (Figure 2C). Together, these findings highlight the combined influence of salinity and pH gradients on the rhizosphere bacterial community of *A. sinensis*.

When interpreting bacterial diversity patterns across sampling sites, it is important to consider combined effects of multiple soil properties, including salinity, alkalinity, and geographic location. Linear regression analysis showed a weak but significant correlation between rhizosphere bacterial community composition and geographic distance (R^2^ = 0.11, *p* < 0.05) (Figure S1), indicating that nearby sites tend to have similar microbial structures. Notably, the three YRD sites in Dongying (Mo_DY, Mi_DY, No_DY) exhibited higher rhizosphere bacterial diversity than other locations. Previous studies show that rhizosphere communities in the YRD are shaped by environmental filtering and plant recruitment, jointly promoting salt-tolerant functional bacteria and niche differentiation [46,47,48,49]. This study supports that view. The dominant bacterial phyla in *A. sinensis* rhizosphere soils were Proteobacteria, Actinobacteriota, Firmicutes, Bacteroidota, and Chloroflexi. Proteobacteria was significantly more abundant in medium-salinity environments and in the YRD, suggesting it plays a key role in *A. sinensis*-associated microbial communities under saline conditions.

### 4.3. Differences in the Composition of Rhizosphere and Endorhizosphere Bacterial Communities and Their Influencing Factors

This study found that bacterial OTU richness and diversity indices (Chao1, Shannon, Simpson) in the rhizosphere of *A. sinensis* were significantly higher than in the endorhizosphere (Table 2), consistent with patterns observed in other plant systems. In terms of composition, Proteobacteria and Actinobacteriota accounted for 30.43% and 18.86% in the rhizosphere, respectively, while Proteobacteria dominated the endorhizosphere at 70.57%, with Actinobacteriota at 16.86% (Figure 3C,D). This mirrors findings in rice, where Proteobacteria comprises 50% on the root surface and up to 75% internally, highlighting its key role in *A. sinensis* ecological adaptation and potential support for survival in specific environments. Linear regression showed the endorhizosphere community, unlike the rhizosphere, was significantly positively correlated with soil nutrients (AP, TN) but not with salinity or pH. Existing studies suggest grass endorhizosphere communities are primarily shaped by host factors—such as root exudates, physiology, and genotype—leading to stable, functionally biased assemblages [13,14]. *A. sinensis* exhibits strong clonal propagation and stabilizes sandy soils via dense stolons and roots, showing promise for restoring degraded coastal or saline–alkali lakefront soils [17]. In saline environments, *A. sinensis* selectively enriches salt-tolerant endophytic microorganisms in its root system, thereby enhancing host stress tolerance through multiple mechanisms, including extracellular polysaccharide production, osmotic regulation, and biological nitrogen fixation.

### 4.4. The Co-Occurrence Patterns of A. sinensis Rhizosphere Microbiome

In saline soils, chemoheterotrophy and aerobic chemoheterotrophy are the dominant functional pathways among prokaryotes [27,50]. Network module analysis identified ME2 and ME9—modules positively correlated with electrical conductivity (EC)—as enriched in chemoheterotrophy-related OTUs in medium-salinity environments. Oceanospirillales, a prominent lineage within the γ-Proteobacteria, is predominantly organoheterotrophic and frequently exhibits halophilic or halotolerant traits, which accounts for its widespread distribution in marine and saline habitats [51]. Multiple Oceanospirillales OTUs were detected within the ME2 module in rhizosphere samples from the Yellow River Delta (YRD), including OTU2042, OTU2583, OTU16692 (Halomonadaceae), OTU21361 (Kangiellaceae), OTU2588, OTU1899 (Nitrincolaceae), and OTU15260 (Pseudohongiellaceae), suggesting their potential involvement in heterotrophic metabolism under elevated salinity in *A. sinensis*. However, previous studies have shown that high salinity exerts inhibitory effects on chemoheterotrophy and aerobic chemoheterotrophy in bacterial communities of rice rhizosphere soil [50]. In this study, the ME1 module, positively correlated with pH and soil elemental composition, exhibited higher abundances of chemoheterotrophy-associated OTUs in no-salt and low-salt sampling sites (Figure 5C). These findings indicate that rhizosphere microorganisms involved in chemoheterotrophy in *A. sinensis* adopt differentiated adaptive strategies in response to varying environmental gradients.

Nitrification—the oxidation of ammonia to nitrite and subsequently to nitrate—is a pivotal process in the nitrogen cycle [52]. At the Yellow River Delta (YRD) saline site (Mo_DY), members of module ME14 were most abundant, particularly ammonia-oxidizing bacteria OTU14871 (Nitrosococcaceae) and OTU2126 (Kiloniellaceae), underscoring the critical role of rhizosphere-associated ammonia-oxidizing bacteria (AOB) in nitrogen cycling within the *A. sinensis*-dominated saline–alkali ecosystem. Furthermore, under low-salt conditions, the rhizosphere microbiota of *A. sinensis* in modules ME1 and ME4 exhibited relatively high abundances of nitrogen-fixing bacteria, such as those belonging to the order Rhizobiales. In low-salinity environments, diverse plant species—including wild soybean (*Glycine soja*) from the Fabaceae family—co-occur with *A. sinensis* to form stable plant communities. Rhizobia associated with wild soybean root nodules demonstrate the capacity to colonize neighboring plants, thereby enhancing their nitrogen acquisition, physiological responsiveness to nitrogen availability, and overall growth performance [53].

### 4.5. Isolation of Salt-Tolerant Rhizosphere Bacteria from A. sinensis

In this study, a plant salt-tolerant strain, *Arthrobacter bergerei* (KF_254744.1), was isolated through a corn irrigation experiment (Figure 6). This strain belongs to the genus Arthrobacter, which is commonly found in extreme environments such as saline–alkali soils, arid regions, and areas contaminated with heavy metals. It exhibits strong environmental adaptability and potential for promoting plant growth. Numerous species within this genus can mitigate the adverse effects of saline–alkali stress on plants through various mechanisms, including the secretion of plant hormones, production of ACC deaminase, exopolysaccharide synthesis, phosphorus solubilization, and nitrogen fixation [54]. Aslam and Ali [55] isolated multiple Arthrobacter strains from the rhizosphere of *Suaeda glauca* and demonstrated that inoculation significantly enhanced plant growth under high-salt conditions, as indicated by increased chlorophyll content, greater aboveground and root biomass, and improved water use efficiency. Pishchik et al. [56] identified drought- and salt-tolerant Arthrobacter strains with plant growth-promoting properties and found that their application in wheat irrigation significantly improved salt–alkali resistance [57]. Inoculated wheat exhibited higher seedling survival rates and biomass accumulation, along with enhanced water use efficiency and osmotic adjustment capacity. Collectively, these findings suggest that the Arthrobacter strain isolated in this study holds significant potential for future applications in halophytes and stress-resistant Poaceae crops.

## 5. Conclusions

This study investigated microbial communities in the endorhizosphere and rhizosphere soils of *Aeluropus sinensis*. The results demonstrate that soil salinity is a key factor shaping the diversity of rhizosphere bacterial communities, with moderate salinity levels enhancing bacterial diversity. Proteobacteria predominate in both root and rhizosphere microbiomes of *A. sinensis* across various habitats. In contrast, the endorhizosphere bacterial community exhibited a significant positive correlation with soil nutrient indicators, including available phosphorus (AP) and total nitrogen (TN), but showed no significant association with soil salinity or pH. The dominant bacterial phyla in *A. sinensis* rhizosphere soils were Proteobacteria, Actinobacteriota, Firmicutes, Bacteroidota, and Chloroflexi. Co-occurrence network analysis revealed that chemoheterotrophic microorganisms in the rhizosphere of *A. sinensis* employ distinct adaptive strategies along environmental gradients, while ammonia-oxidizing bacteria (AOB) likely play a potential role in nitrogen cycling within the Yellow River Delta (YRD) saline–alkaline freshwater ecosystem. Furthermore, this study successfully isolated a salt-tolerant plant-associated bacterial strain, *Arthrobacter bergerei* (KF_254744.1), through a maize irrigation experiment.

## Figures and Tables

**Table 1:** Sampling site information of *Aeluropus sinensis*.

Name	Collection Site	Latitude	Longitude	Altitude
Mo_DY	Shandong, Dongying, Hekou	38°3′5″ N	118°45′2″ E	−4 m
Mo_HB	Hebei, Hengshui, Jizhou	37°32′48″ N	115°31′20″ E	22 m
Mi_NX	Ningxia, Shizuishan, Pingluo	38°49′58″ N	106°23′1″ E	1099 m
Mi_DY	Shandong, Dongying, Kenli	37°45′43″ N	119°5′52″ E	2 m
Mi_TJ	Tianjin, Binhai NewArea	38°39′52″ N	117°32′17″ E	9 m
No_JS	Jiangsu, Yancheng, Sheyang	33°39′30″ N	120°26′52″ E	5 m
No_DY	Shandong, Binzhou, Wudi	38°15′36″ N	117°51′58″ E	27 m

Note: Altitude refers to the height difference from mean sea level (MSL).

**Table 2:** The α diversity index of rhizosphere and endophytic bacterial communities in *Aeluropus sinensis*.

Name		OTUs	Chao1	Shannon	Simpson
Rh	Mo_DY	3706 ± 39.39 A	4795.08 ± 56.96 A	6.69 ± 0 AB	0.007 ± 0 B
	Mo_HB	2623.33 ± 196.66 C	3681.83 ± 117.38 B	5.61 ± 0.2 C	0.017 ± 0.2 B
	Mi_NX	2946.66 ± 62.97 BC	3820.55 ± 63.68 B	6.66 ± 0.02 AB	0.003 ± 0.02 B
	Mi_DY	4068.33 ± 157.82 A	5255.9 ± 152.49 A	7.03 ± 0.01 A	0.003 ± 0.01 B
	Mi_TJ	2981 ± 197.3 BC	3733.99 ± 202.79 B	6.34 ± 0.02 B	0.007 ± 0.02 B
	No_JS	2080.33 ± 529.13 D	3044.07 ± 358.37 C	4.11 ± 0.59 D	0.12 ± 0.59 A
	No_DY	3153 ± 89.71 B	4026.94 ± 25.99 B	6.74 ± 0 AB	0.003 ± 0 B
Ri	Mo_DY	138.66 ± 57.5 b	168.49 ± 36.58 b	3.26 ± 0.26 bc	0.065 ± 0.01 b
	Mo_HB	476 ± 34.11 a	725.46 ± 16.56 a	4.54 ± 0.22 a	0.036 ± 0.01 b
	Mi_NX	234.33 ± 40.12 b	277.64 ± 31.36 b	3.75 ± 0.19 ab	0.067 ± 0.01 b
	Mi_DY	243 ± 62.52 b	303.56 ± 50.95 b	3.88 ± 0.21 ab	0.044 ± 0 b
	Mi_TJ	211.33 ± 85.12 b	297.37 ± 74.2 b	3.43 ± 0.49 bc	0.078 ± 0.03 b
	No_JS	190 ± 116.5 b	314.09 ± 91.39 b	2.45 ± 0.48 c	0.228 ± 0.06 a
	No_DY	184.33 ± 18.14 b	261.02 ± 36.54 b	3.4 ± 0.05 bc	0.06 ± 0 b

Note: Uppercase letters (A, B, C, D) indicate statistically significant differences (*p* < 0.05) in α-diversity indices among rhizosphere samples; lowercase letters (a, b, c) indicate statistically significant differences (*p* < 0.05) in α-diversity indices among endorhizal samples. Rh: rhizosphere, Ri: endorhizosphere.

**Table 3:** The membership function value based on physiological indicators.

Strain Name	Fresh Weight	Dry Weight	Chlorophyll	Proline	Sort
Aes1	0.224	0.128	0.352	0.813	7
**Aes2**	**0.557**	**0.347**	**0.497**	**0.626**	**1**
Aes3	0	0	0.277	0.845	11
Aes4	0.503	0.404	0.557	0.545	2
Aes5	0.34	0.203	0.388	1	3
Aes6	0.306	0.172	0.149	0.976	5
Aes7	0.326	0.306	0.454	0.457	6
Aes8	0.394	0.222	0.006	0.852	8
Aes9	0.102	0.23	0.098	0.791	10
Aes10	0.061	0.243	0	0.996	9
Aes11	0.231	0.333	0.593	0.523	4

Note: The bolded font highlights the information of the selected salt-tolerant strains.

## Data Availability

The data presented in this study are openly available in [NCBI database] at [https://www.ncbi.nlm.nih.gov/, accessed on 10 January 2025], reference number [PRJNA1273014].

## Supplementary Materials

The following supporting information can be downloaded at https://www.mdpi.com/article/10.3390/microorganisms14010165/s1, Figure S1: Linear regression analysis of Shannon distance and geographic distance in both the rhizosphere (A) and endorhizal tissues (B). Rhizosphere soil bacteria are represented by red dots, while rhizosphere tissue bacteria are represented by blue dots; Figure S2: The Colony phenotypic traits of 11 bacterial isolates were obtained from the rhizosphere soil of A. sinensis collected from the Mo_DY site. Aes2 colonies exhibit cream-yellow coloration, round shape, smooth surface, and opacity; Figure S3: Comparative analysis of growth and physiological traits of maize seedlings under different bacterial inoculation treatments under control and salt stress conditions. The differences in phenotypic and physiological indicators between the control group and the salt treatment group were analyzed using one-way ANOVA. The p-values are presented as asterisks above the box plots. **** indicates *p*-value < 0.001, *** indicates *p*-value < 0.005, ** indicates *p*-value < 0.01, * indicates *p*-value < 0.05, and ns indicates there is no significant difference; Table S1: OTU information contained in the modules of the co-occurrence network.
