# Supplementary figures and images for: Bacterial Community Structure and Environmental Adaptation in the Endorhizosphere and Rhizosphere Soils of Aeluropus sinensis from Saline Lands Across Coastal and Inland Regions of China
Source: Microorganisms. 2026 Jan 12;14(1):165. doi: 10.3390/microorganisms14010165 (PMC12843883; doi:10.3390/microorganisms14010165)

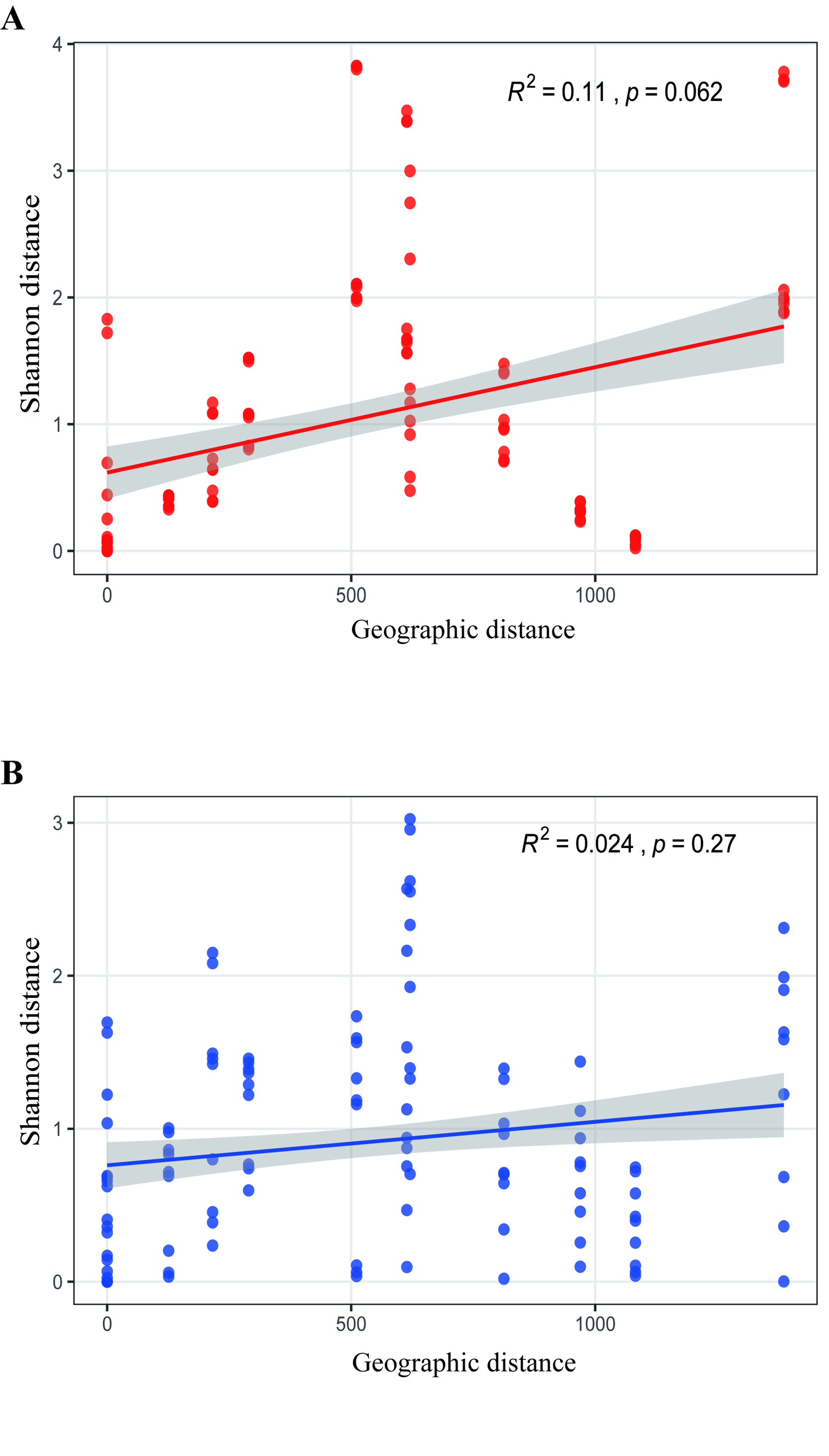

Supplement: Supplementary file 1 [file microorganisms-14-00165-s001.zip › Figure S1.jpg]

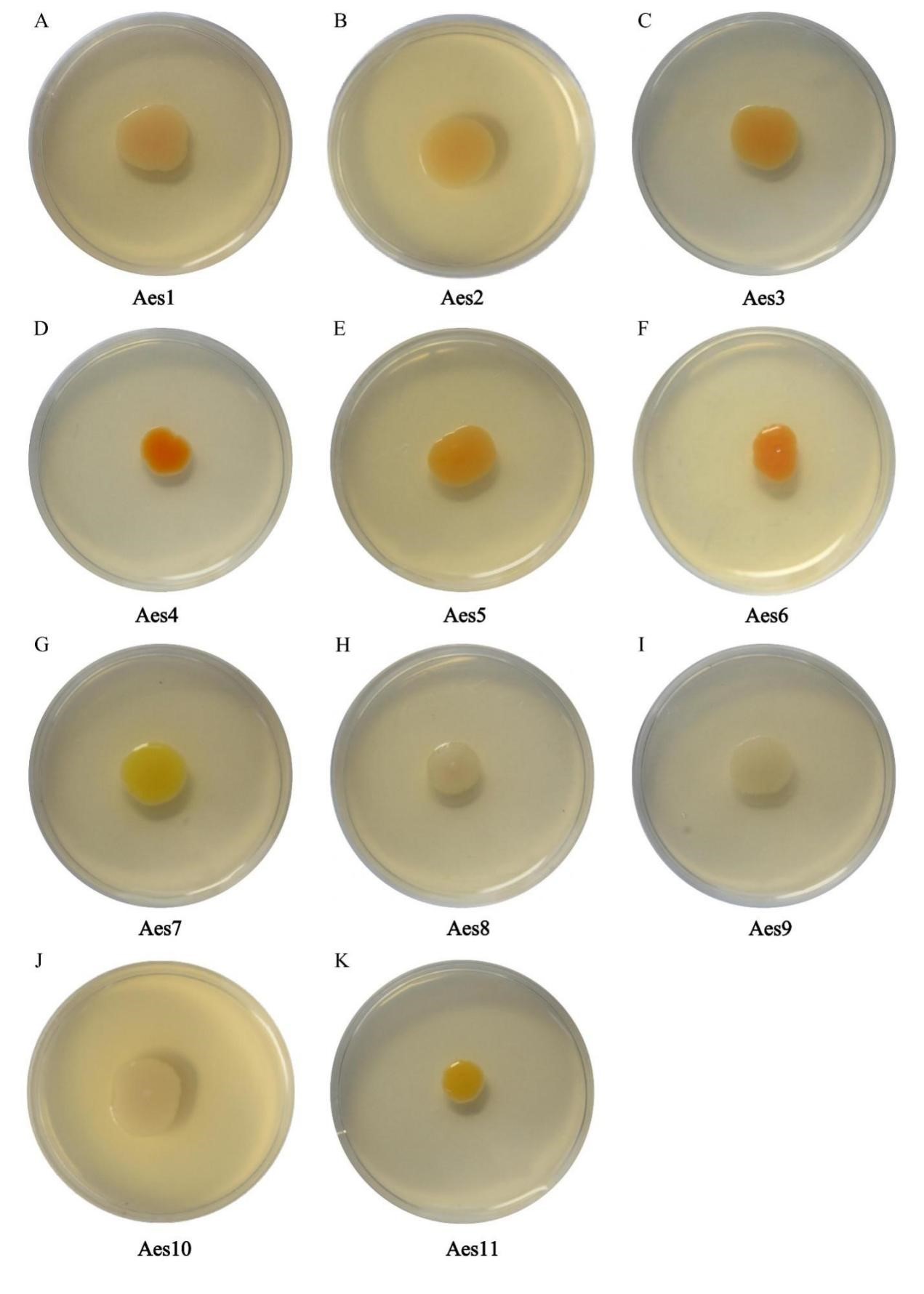

Supplement: Supplementary file 1 [file microorganisms-14-00165-s001.zip › Figure S2.jpg]

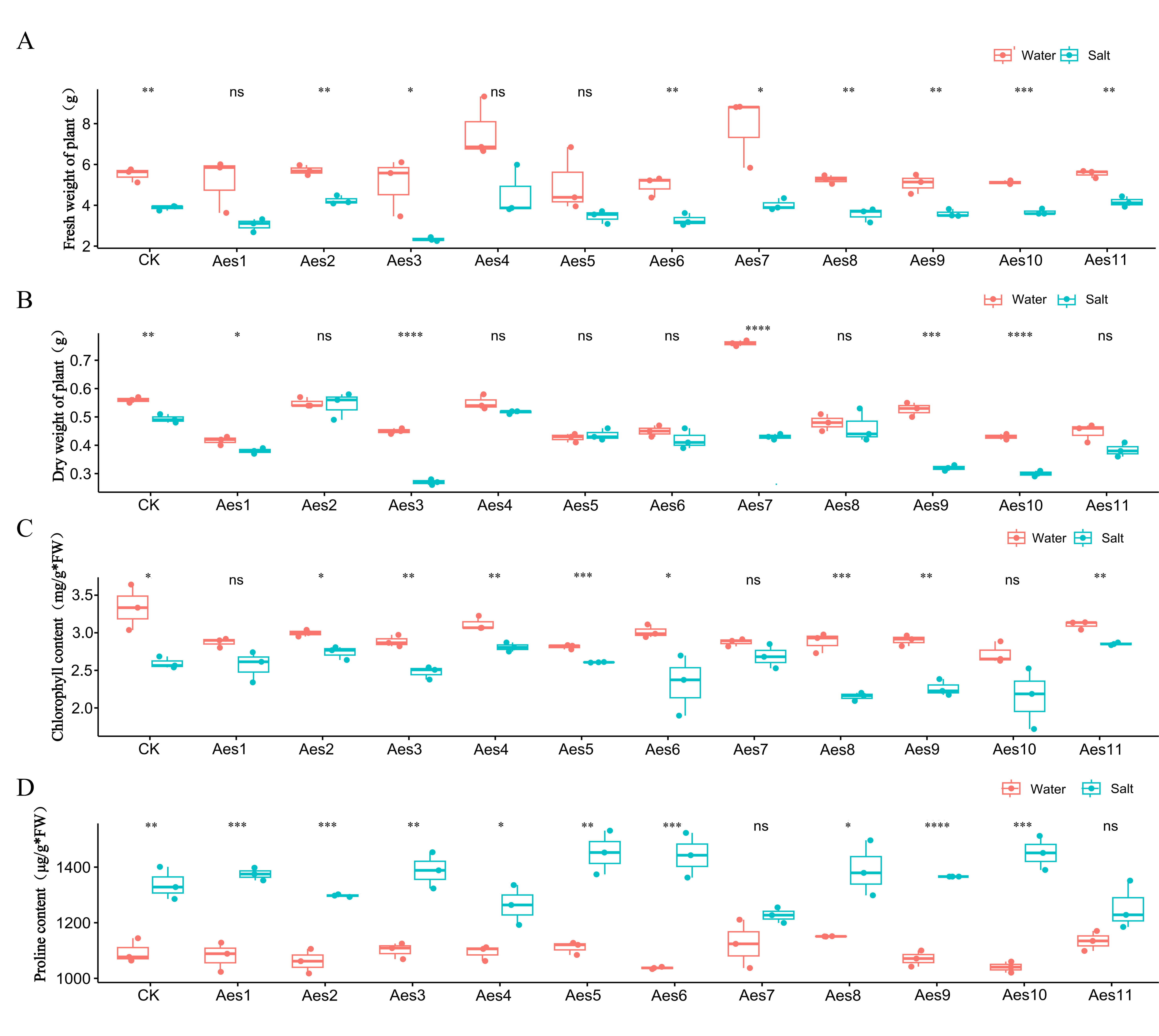

Supplement: Supplementary file 1 [file microorganisms-14-00165-s001.zip › Figure S3.jpg]
